# Relativistic-intensity near-single-cycle light waveforms at kHz repetition rate

**DOI:** 10.1038/s41377-020-0280-5

**Published:** 2020-03-23

**Authors:** Marie Ouillé, Aline Vernier, Frederik Böhle, Maïmouna Bocoum, Aurélie Jullien, Magali Lozano, Jean-Philippe Rousseau, Zhao Cheng, Dominykas Gustas, Andreas Blumenstein, Peter Simon, Stefan Haessler, Jérôme Faure, Tamas Nagy, Rodrigo Lopez-Martens

**Affiliations:** 1 Laboratoire d’Optique Appliquée, CNRS, Ecole Polytechnique, ENSTA Paris, Institut Polytechnique de Paris, 181 chemin de la Hunière et des Joncherettes, 91120 Palaiseau, France; 2 Ardop Engineering, Cité de la Photonique, 11 Avenue de la Canteranne, bât. Pléione, 33600 Pessac, France; 3 Laser-Laboratorium Göttingen e.V., Hans-Adolf-Krebs-Weg 1, 37077 Göttingen, Germany; 4 Max Born Institute for Nonlinear Optics and Short Pulse Spectroscopy, Max-Born-Strasse 2A, 12489 Berlin, Germany

**Keywords:** Ultrafast lasers, High-field lasers, Plasma-based accelerators

## Abstract

The development of ultra-intense and ultra-short light sources is currently a subject of intense research driven by the discovery of novel phenomena in the realm of relativistic optics, such as the production of ultrafast energetic particle and radiation beams for applications. It has been a long-standing challenge to unite two hitherto distinct classes of light sources: those achieving relativistic intensity and those with pulse durations approaching a single light cycle. While the former class traditionally involves large-scale amplification chains, the latter class places high demand on the spatiotemporal control of the electromagnetic laser field. Here, we present a light source producing waveform-controlled 1.5-cycle pulses with a 719 nm central wavelength that can be focused to relativistic intensity at a 1 kHz repetition rate based on nonlinear post-compression in a long hollow-core fiber. The unique capabilities of this source allow us to observe the first experimental indications of light waveform effects in laser wakefield acceleration of relativistic energy electrons.

## Relativistic optics: High intensity ultra-short fast-repeating light pulses

A pioneering laser source combines extremely high intensity and fast-repeating pulses with ultra-short pulse duration, opening new opportunities in the field of research and technology called relativistic optics. This requires lasers that are sufficiently intense to accelerate particles such as electrons to close to light speed, when effects of relativity theory become increasingly significant. Marie Ouillé and colleagues at the CNRS Laboratoire d’Optique Appliquée in France, with co-workers in Germany, combined laser sources with light compression and manipulation methods to generate relativistic intensity pulses almost as short as a single cycle of the light wave. They say their system is currently the only light source capable of achieving pulses shorter than four femtoseconds combined with peak powers up to 1 terawatt. The researchers also demonstrated precise control over the fine structure of the light pulses.

## Introduction

Waveform-controlled few-cycle laser pulses are formidable optical tools that unite temporal finesse on the attosecond time scale with ultra-high electromagnetic field strengths. Laser transients approaching a single carrier wave period have been available for more than a decade with sufficient average (<1 W) and peak power (≤0.1 TW) to drive laser–atom interactions in the strong-field regime, where a laser electric field of ≳10^10^ V/m starts overcoming that binding valence electrons to the nucleus. The temporal precision afforded by carrier-envelope phase (CEP) control of these pulses helped pave the way for attosecond science^1–3^, which continues to further our understanding of fundamental ultrafast many-body quantum dynamics.

With laser electric field strengths of ≳10^13^ V/m, the field-driven quiver energy of electrons exceeds their rest mass energy ($m_{\mathrm{e}}c^2 \approx 0.5$ MeV), that is, electrons reach relativistic velocities within one laser period. Here, the magnetic component of the Lorentz force becomes equally important or even dominates the electric component, and the electron dynamics become highly nonlinear as a function of the laser field. Laser–matter interaction in this relativistic regime^4–6^ is at the forefront of contemporary physics, uniting highly complex plasma dynamics with the highest achievable laser field strengths. This launched the development of highly promising secondary energetic particle and light sources, such as laser-plasma electron accelerators^7^ and plasma mirror-based attosecond extreme ultraviolet sources^8^.

Generating CEP-controlled few-cycle pulses capable not only of driving such relativistic laser–plasma interactions but also of steering these extreme light forces with attosecond precision has been a long-standing challenge. This is chiefly motivated by the power scaling of laser wakefield acceleration (LWFA) of relativistic electrons^9^ and the generation of intense isolated attosecond pulses from relativistic plasma mirrors^10^ or thin transmission targets^11^. From a more fundamental standpoint, few-cycle pulses exhibit the fastest intensity gradients and enable the isolation of electromagnetic field-driven processes from cycle-averaged intensity effects.

Recent developments have led to a significant increase in the power generated by few-cycle laser pulse sources. Broadband optical parametric chirped pulse amplifiers (OPCPAs) have succeeded in scaling up the peak power of few-cycle pulses to the multi-TW range^12–14^. However, due to their need for large pump energies of several hundreds of mJ, these systems usually operate only at low repetition rates (10 Hz), which denies proper CEP stabilization. Nonetheless, because of their inherently high temporal contrast, OPCPA-based sources have recently enabled few-cycle-driven relativistic laser–plasma interactions at a 10-Hz repetition rate, demonstrating relativistic surface high-harmonic generation with sub-2-cycle^13^ and sub-3-cycle^10,14,15^ pulses, as well as LWFA with 3-cycle pulses^16^.

Efforts to scale the average power have led to a >200 W sub-2-cycle pulse source^17^ based on high-repetition-rate (100 kHz) ytterbium-based fiber chirped pulse amplifier technology combined with hollow-core fiber (HCF) nonlinear post-compression^18^. While the achievable compression factors have increased 33-fold^19^, CEP locking has yet to be implemented. On the other hand, progress in high-repetition-rate pump laser technology and passively CEP-stable seed generation^20^ for TW-class OPCPAs has recently led to the production of ~10 W average power sub-3-cycle pulses with a stable CEP^21^, but their capabilities for high-field applications have yet to be harnessed.

Novel schemes for the generation of terawatt or even petawatt few-cycle pulses have been proposed, and recently, the first experimental proof-of-principle demonstrations have been reported for thin-film compression of top-hat pulses^22^ as well as frequency downshifting of intense optical pulses into the mid-infrared via plasma-based photon deceleration^23^.

A competing approach for TW-level sub-2-cycle pulse generation relies on the power scaling of HCF-based post-compression of pulses produced by Ti:sapphire chirped pulse amplification (CPA) systems^24^. This approach was shown earlier to provide 2-cycle pulses at 1 TW peak power^25^, albeit without CEP control. The first system following this approach and uniting multi-mJ pulse energy, sub-2-cycle duration, and CEP locking^26^ was the precursor to the system described here. In an intermediate development stage with increased energy from the laser amplifier chain and improved vacuum integration of the HCF but lacking CEP control due to insufficient pulse energy stability, the system was the first to drive LWFA to relativistic electron energies at a kHz repetition rate^9^.

Here, we present our most advanced setup as the result of a large engineering effort resulting in significant performance improvements: the pulses set a new duration record (1.5 cycles) for TW peak power pulses, and the pulse stability (CEP, energy, spatial, and spectral) fulfills the requirements for relativistic-intensity laser–matter experiments. Combining the kHz repetition rate, high temporal contrast ratio, and achievable ultra-high intensity on the target, this system is now uniquely suited to drive relativistic-intensity light–matter interactions with sub-cycle time control over the driving light waveform. Here, we describe this unique light source and the first experimental indications of CEP effects in relativistic LWFA driven in the near-single-cycle regime.

## Results

### Power-scaled hollow fiber compressor

**Fig. 1:**
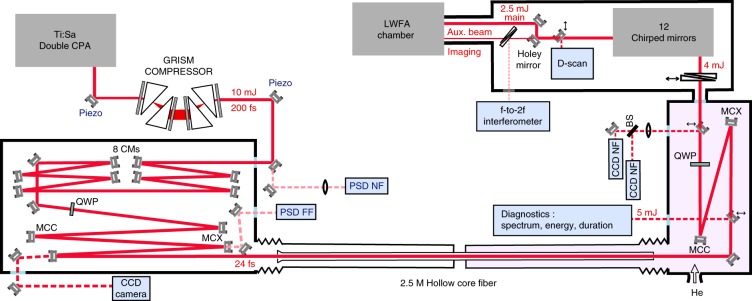
Schematics of the vacuum-integrated stretched flexible hollow fiber pulse compressor setup. PSD photosensitive detector, NF near field, FF far field, piezo piezo-driven mirror mounts, MCX convex mirror, MCC concave mirror, QWP quarter-wave plate

The fully vacuum-integrated post-compressor setup is shown in detail in Fig. 1. The seed laser is a Ti:sapphire double CPA chain delivering pulses with 10 mJ energy (shot-to-shot stability of <0.3% root mean square (r.m.s.) over hours) at a 1-kHz repetition rate with a temporal contrast of >10^10^ at ≈−10 ps before the pulse peak^27^. Pulses from the CPA are first only partially compressed to ≈200 fs in air to prevent nonlinear beam degradation in the entrance window of the vacuum beamline, which would significantly reduce the coupling efficiency into the HCF^26^. Final compression to the Fourier transform-limited duration of 24 fs is achieved after eight highly dispersive chirped mirrors under vacuum introducing ≈−2000 fs^2^ group delay dispersion (GDD).

The double CPA seed laser has been improved compared to that in refs. ^9,27^. Its nonlinear crosspolarized wave (XPW) contrast filter and low stretching factor lead to a significant B-integral accumulated through the amplifier chain, and thus, noise is coupled from intensity to phase. CEP locking thus first requires excellent pulse energy stability. This has been achieved by replacing the pump laser of the front end (to an Ascend 40 by Spectra Physics, Santa Clara, California), optimizations of the power amplifier layout, and enhanced enclosures of the laser and all pump beams to protect from air movement. Other improvements include the configuration of the low-jitter mode of the two acousto-optical programmable dispersive filters in the seed laser chain.

The post-compression stage is based on self-phase modulation (SPM) in a gas-filled HCF, a now widely used technique^18^ that has the advantage of producing well-compressible broadband pulses with excellent beam profiles and spatially homogeneous spectra^28^. We implemented a combination of three strategies to scale this technique to the ≈0.4 TW peak power of our CPA chain. First, we exploit stretched flexible HCF technology enabling an arbitrary waveguide length without degradation of the waveguiding properties^29,30^. The HCF dimensions were scaled to 2.5 m length and 536 μm inner diameter. A conical glass taper is coaxially installed at the HCF entrance to create very robust protection against damage due to slight misalignments or pedestals in the spatial beam profile. The beam is focused into the fiber using a reflective mirror telescope with an effective focal length of ≈4.2 m, and the optimal coupling into the fiber is maintained with active beam-pointing stabilization to ensure long-term stability. Second, high-purity helium gas is differentially pumped through the HCF, thus forming a stable pressure gradient across the waveguide^31^. The pressure gradually increases from <1 mbar at the fiber entrance to the static filling pressure of the output chamber up to 2 bar. This prevents undesirable nonlinear phenomena around the fiber entrance and enhances the coupling efficiency and stability. Furthermore, the increasing pressure counteracts the decreasing nonlinearity due to propagation losses inside the fiber. Third, both multi-photon ionization and self-focusing are further mitigated by using circular polarization^32,33^, which also reduces losses and instabilities due to cycling of energy between fiber modes^34^. Two broadband quarter-wave plates are therefore placed before and after the HCF to switch the laser polarization between linear and circular.

**Fig. 2:**
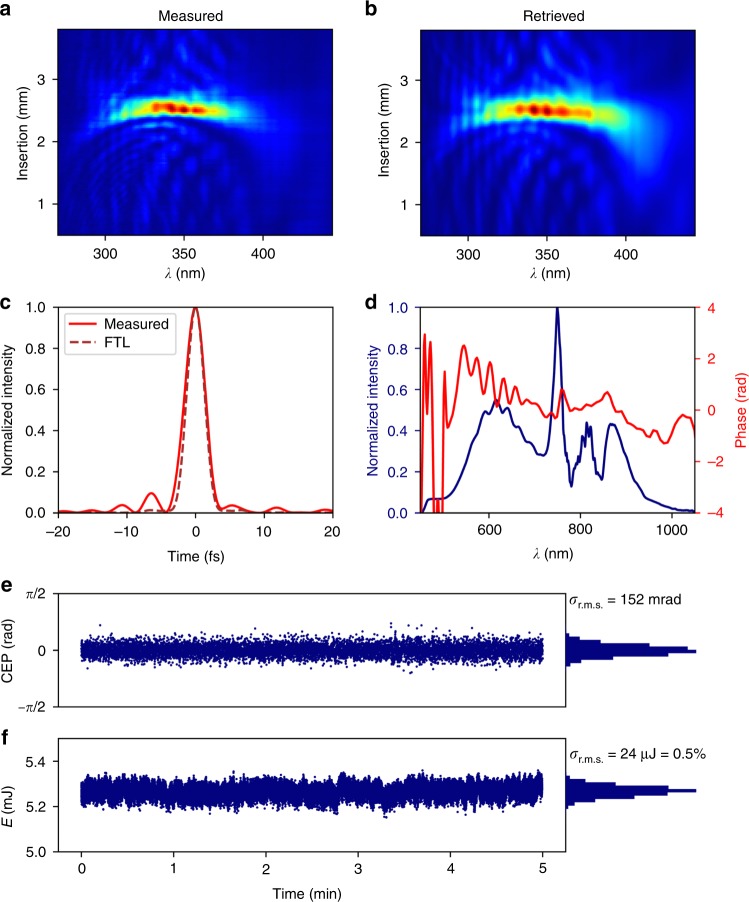
D-scan measurement for 1.3 bar helium pressure. Measured (**a**) and retrieved (**b**) d-scan traces. Reconstructed temporal profile (**c**): the retrieved temporal pulse profile is shown in red (3.4 fs FWHM). The ideal Fourier transform-limited shape (2.9 fs FWHM) is indicated by the brown dotted line. Measured spectral intensity and phase (**d**). Relative CEP stability (each point averaged over 30 shots) (**e**) and pulse energy at the fiber exit (**f**), both measured over 5 min

In the output chamber, two insertable mirrors can send the beam to diagnostics for position, spatial profile, spectrum and pulse energy. Two active mirrors then allow for alignment onto the near- and far-field references thus obtained. A 3-mm-thick fused silica window separates the helium-filled output chamber from the vacuum beamline downstream. The beam first propagates through a pair of motorized fused silica wedges. The positive GDD induced by SPM in the fiber as well as the propagation through the quarter-wave plate and the window is slightly over-compensated by six pairs of highly dispersive (−40 fs^2^ each) double-angle chirped mirrors (PC70, Ultrafast Innovations, Garching, Germany), and the wedge position is tuned for fine adjustment of the GDD leading to the optimum pulse compression. A dispersion (d)-scan device (Sphere Ultrafast Photonics, Porto, Portugal) placed under vacuum serves as a precise temporal measurement immediately before the experimental chamber with controllable dispersion provided by precise insertion of one of the wedges. With 1.3 bar helium pressure in the output chamber, the minimum achievable full-width at half-maximum (FWHM) pulse duration of our system is 3.4 ± 0.1 fs (see Fig. 2c). This duration is close to the Fourier limit of 2.9 fs (with 60% higher peak power) supported by the broadened spectrum.

This is a remarkable result indicating accurate compensation of not only the GDD but also the third-order dispersion (TOD), to which compression in the sub-two-optical-cycle regime is extremely sensitive^35–38^. As a consequence, venting the d-scan chamber with air is sufficient to spoil the compression shown in Fig. 2, and the optimal wedge position leaves a significant negative TOD on the compressed pulses, similar to the result reported in ref. ^39^. This is due to the different GDD/TOD ratio of air compared to that of the fused silica wedges.

The remaining room for improvement on the result shown in Fig. 2 is due to two imperfections in the compression. The first and minor imperfection is a very small remaining negative TOD of a few fs^3^. The second and dominant imperfection is the phase distortion introduced by the chirped mirrors on the blue end of the spectrum. The phase oscillations between 700 and 550 nm reduce the energy contained in the main peak, and smoothing them (possible by using complementary-pair CMs instead of double-angle CMs) combined with a TOD of −5 fs^3^ would increase the peak power by 15% and reduce the pulse duration to 3.2 fs. To further approach the Fourier limit, the uncontrolled phase below 520 nm would need to be smoothed and flattened, which would require a more advanced and, to our knowledge, not yet available CM design. This would decrease the pulse duration to 3.2 fs and increase the peak power by 20%.

Energy measurements performed for 1.3 bar of helium with a single-shot energy detector (noise level ≈10 μJ) yield an excellent pulse-to-pulse stability of ≈0.4% rms and typical pulse energies of 4.5–5 mJ immediately after the fiber, 3.5 mJ after the chirped-mirror compressor at the entrance of the d-scan device and 2.5 mJ on the target in the LWFA chamber. Losses along the beamline are due to the wave plate, wedges (≈5%), CMs (≈5% from all 12 CMs) and transport mirrors (≈1.5% per mirror).

CEP stabilization of the system is based on a home-made f-to-2f interferometer. As shown in Fig. 1, the f-to-2f signal is generated using the reflection from the front face of a thin wedge pair placed in the beam path of an auxiliary beam created with a holed mirror and used for plasma imaging^9^. The derived error signal then modulates the phase offset of the oscillator locking electronics to correct for slow CEP drifts accumulated through the laser chain. As shown in Fig. 2e, the residual CEP noise of the system, measured in-loop over 5 min, is ≈150 mrad r.m.s.

### Pulse duration tuneability

**Fig. 3:**
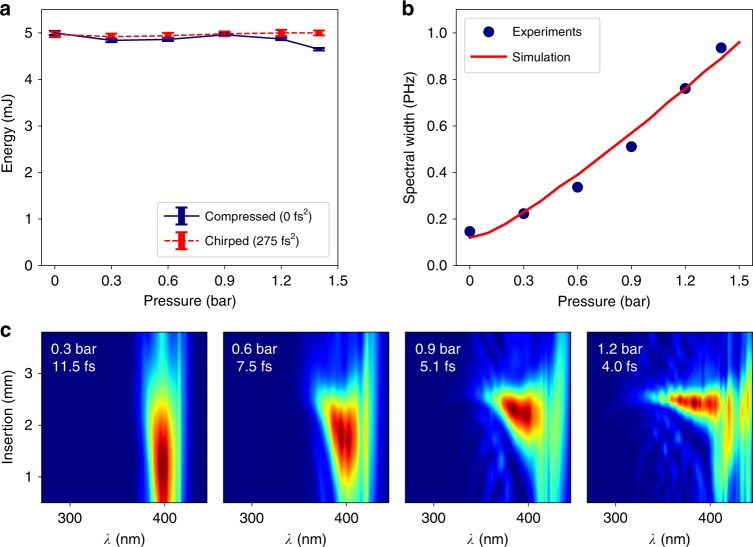
**a** Evolution of the output energy with the gas pressure for a compressed pulse (blue) and a positively chirped pulse (red) showing that we do not have ionization-induced losses, except at the highest pressures. **b** The spectral width evolution while tuning the helium pressure in the output chamber (blue dots) is in good agreement with numerical simulations taking only Kerr nonlinearities into account (red line). **c** D-scan traces for different pressure values showing the pulse duration tunability of the laser

Compression data for different gas pressures (Fig. 3) show that our HCF post-compression stage is adequately energy scaled, that is, self-focusing and ionization-induced effects are avoided: the fiber transmission is the same for a Fourier-limited input pulse as for a positively chirped (+275 fs^2^) input pulse; the fiber transmission remains the same over the complete helium pressure range used. Only at the highest pressure of 1.4 bar, which is above our usual working range, does the transmission start to drop. Finally, as shown in Fig. 3b, the measured spectral broadening follows the helium pressure *p* as expected for purely SPM-induced broadening. We compare the experimental spectral widths, defined as twice the r.m.s. bandwidth ${\upsigma}_{\upomega} = \sqrt { {\left\langle {{\upomega}^2} \right\rangle - \left\langle \omega \right\rangle ^2}}$, to values obtained from a numerical solution of the one-dimensional generalized nonlinear Schrödinger equation^40,41^ for a Kerr nonlinearity^42^. These simulations start from an experimentally measured input pulse obtained from a Wizzler measurement^43^ (Fastlite, Antibes, France) located after the diagnostics port of the output chamber (cf. Fig. 1) for an evacuated HCF and describe dispersion, SPM and self-steepening. The latter significantly modulates SPM-induced spectral broadening for input pulses as short as ours. The pressure gradient along an HCF of length *L* = 2.5 m was modeled as $p(z) = p_{L}\sqrt {z/L}$, where *p*_*L*_ is the pressure at the HCF exit located at *z* = *L*. Neither ionization-induced effects nor spatial effects had to be included in these 1D simulations to obtain satisfactory agreement with the experimental spectral width and shape. Note that the functional shape of the pressure dependence remains very close to that derived for pure SPM and Fourier transform-limited Gaussian input pulses^44^.

An interesting consequence of this well-scaled post-compression stage is that the pulse duration can be easily tuned from 25 fs down to 3.5 fs by simply varying the gas pressure and adjusting the dispersion while keeping a constant output pulse energy (see Figs. 2 and 3c) and a very similar spatial beam profile imposed by the waveguide.

### CEP dependence of relativistic LWFA

**Fig. 4:**
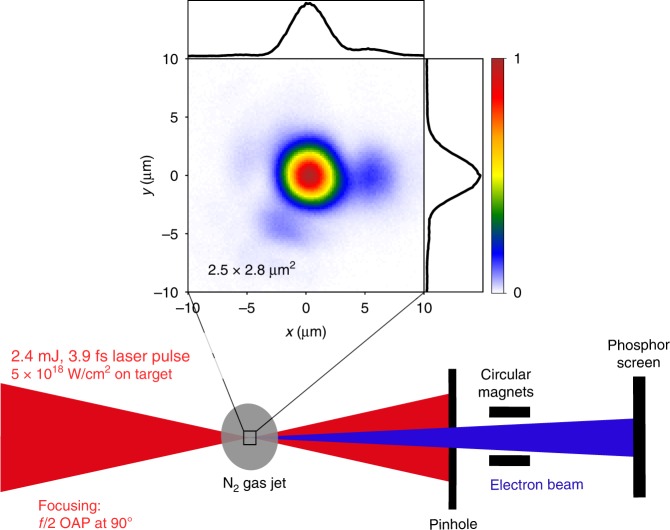
Experimental setup for electron acceleration (top view) and picture of the laser focal spot with size 2.5 × 2.8 μm^2^ FWHM, resulting in intensities of ≈5 × 10^18^ W/cm^2^

As a demonstration of the excellent spatiotemporal quality of our system, we now focus on its application to LWFA in the relativistic-intensity regime^45^. The experimental setup for LWFA is shown in Fig. 4. LWFA of electrons driven by near-single-cycle pulses was demonstrated with a similar setup but without CEP stability^9,46^. The beam is focused into a continuously flowing microscale supersonic nitrogen gas jet using a 90° off-axis *f*/2 parabola. We obtain a near-Gaussian 2.5 × 2.8 µm^2^ (FWHM) focal spot (see Fig. 4), corresponding to an on-target peak intensity of ≈5 × 10^18^ W/cm^2^ with 2.4 mJ on the target (the relativistic limit for which the normalized vector potential reaches *a*_0_ = 1 is ≈2.6 × 10^18^ W/cm^2^ at 719 nm central wavelength). More details on the electron detection system can be found in ref. ^46^.

In these experiments, the laser pulse envelope drives the wakefield via the ponderomotive force. Electrons are injected into the wakefield in a process known as ionization injection^47,48^. In ionization injection, electrons are born at the peak of the laser electric field and subsequently injected and accelerated into the wakefield accelerating structure. This injection process heavily depends on the CEP because the laser phase determines the initial conditions of electrons in the longitudinal phase space, which eventually impacts their final momenta^49^. More precisely, the CEP controls (i) the amplitude of the most intense laser half-cycle and therefore the number of injected electrons and (ii) the exact initial conditions of the trapped electrons, which impact the final electron energies and angles. Observing such CEP effects in an experiment is rather challenging because the CEP varies rapidly during laser propagation since the laser phase velocity (*v*_φ_) and group velocity (*v*_g_) are different in the plasma. Typically, the CEP spans 2*π* over the phase slippage length *L*_2π_ = *λ*_c_/(*v*_φ_ − *v*_g_) ≈ *λn*_c_/*n*_e_, where *n*_e_ and *n*_c_ are the electron plasma density and critical density, respectively. In our typical LWFA experiments^46^, *n*_e_/*n*_c_ ≈ 0.1 and *L*_2*π*_ ≈ 8 μm, that is, ≈10% of our typical target widths. Therefore, the effect of the CEP on electron injection is significant only if the injection length is smaller than *L*_2*π*_, which therefore requires a very localized injection and places stringent demands on the stability of all other pulse parameters in space, time and energy.

Using the same gas density as in our earlier experiments, *n*_e_/*n*_c_ ≈ 0.1, we obtain the same 6-MeV electron peak energies with the upgraded driver laser, but do not observe any influence of the CEP. Therefore, for the experiment presented here, we used a target with *n*_e_ ≈ 2 × 10^19^ cm^−3^, that is, *n*_e_/*n*_c_ ≈ 0.01, which relaxes the difficulty posed by *L*_2*π*_ at the price of reduced achievable electron energies.

**Fig. 5:**
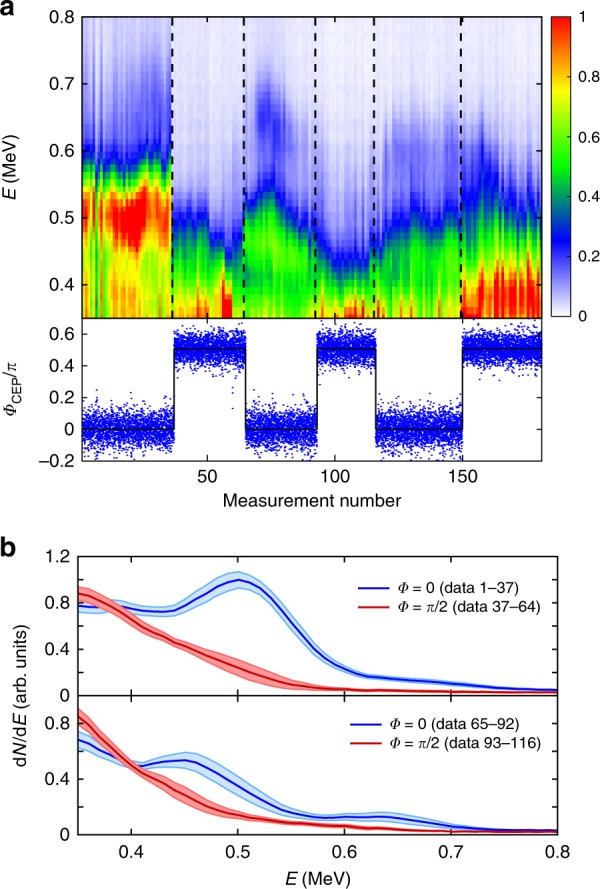
**a** Top: Electron spectra d*N*/d*E* (arb. units) obtained while varying the CEP of the accelerating laser pulse (each spectrum was obtained by averaging over 500 laser shots). Bottom: The measured CEP (blue dots) and the command CEP values sent to the feedback loop (black line). **b** Averaged spectra for the first (top) and second (bottom) CEP cycles. The solid dark lines represent the average spectra, whereas the light blue and red areas indicate the standard deviation due to spectral fluctuations

Under these conditions, a correlation between rapid CEP changes and rapid changes in the electron energy distribution could be observed for the first time. Figure 5a shows a cascade plot of successive electron spectra recorded as the CEP was cycled between 0 and *π*/2. Note that the absolute value of the CEP is not measured, and only the relative changes are known. For a relative CEP of 0, the spectrum exhibits a peak close to 0.5 MeV, and the distribution tail displays another smaller feature at 0.65 MeV. These features disappear when the CEP is shifted by *π*/2. While the effect of the CEP is initially clear (data 1 to 125), it should be noted that the difference tends to wash out towards the end of the scan. The high nonlinearity of the relativistic laser–plasma interaction makes it likely that even small fluctuations or drifts of laser properties or the plasma density profile wash out or outweigh the pure CEP effect.

Figure 5b shows averaged spectra for the first two CEP cycles with a clear correlation and compares the results for relative CEP values of 0 and *π*/2. The CEP effect clearly outweighs that of the spectral fluctuations, as it is significantly larger than the standard deviation of the spectra. These results constitute the first observation of CEP effects in underdense relativistic laser–plasma interactions. However, systematic control of the electron spectra using the CEP has not yet been demonstrated, and future work will focus on enhancing the stability and robustness of this process.

## Discussion

Our laser system delivers pulses combining high peak power up to 1 TW, near-single-cycle pulse duration (3.4 fs or 1.5 cycles at 719 nm central wavelength) and excellent beam quality. Due to the quasi-instantaneous interaction with helium in the HCF post-compressor stage, the high temporal contrast on the picosecond time scale is inherited directly from the double CPA seed laser. This system is currently the only terawatt light source capable of generating sub-4-fs pulses. Furthermore, the output exhibits the necessary spectral, energy and CEP stability required for the investigation of relativistic-intensity laser–matter interactions on a sub-light-cycle time scale. The unique capabilities of the system are demonstrated by the observation of the first experimental indications of CEP effects in LWFA.

### Materials and methods

The Ti:sapphire double CPA seed laser has the following structure: the commercial CPA front end (Femtopower Compact Pro CE Phase, Femtolasers, Vienna, Austria), including a first acousto-optical programmable dispersive filter (low-jitter Dazzler HR800, Fastlite, Antibes, France) and pumped by a frequency-doubled Q-switched Nd:YLF laser (Ascend 40, Spectra Physics, Santa Clara, California), delivers 29-fs pulses with 1.3 mJ energy at a 1 kHz repetition rate and a residual CEP noise of ≈100 mrad (in-loop, averaged over 30 shots). These pulses are sent through an XPW contrast filter^50^ including a Glan polarizer with ~10^4^ extinction ratio, which produces ≈10-fs pulses of ≈200 μJ energy with a temporal contrast of 10^11^. The pulses are then stretched to 45 ps by propagation through 75 cm of SF57 before a double pass through a second low-jitter Dazzler (HR45, Fastlite). The resulting amplitude and phase-shaped seed pulses of ≈20 μJ energy are then sent through a commercial 6-pass amplifier (Femtolasers), boosting the pulse energy to ≈4.5 mJ, followed by a home-built 2-pass amplifier that brings the pulse energy up to ≈13 mJ. These two amplification stages are pumped by frequency-doubled Q-switched Nd:YLF lasers (DM30 and DM50, respectively, Photonics Industries, Long Island, New York). After a GRISM compressor (Fastlite) and subsequent set of eight highly dispersive (−275 fs^2^ each) chirped mirrors (HD58, Ultrafast Innovations, Garching, Germany) under vacuum, the pulses are 24 fs long and have 10 mJ energy at a 1 kHz repetition rate.

These pulses are then converted to circular polarization by a superachromatic quarter-wave plate (Bernhard Halle, Berlin, Germany). The same wave plate converts the pulses back to linear polarization after the HCF. A telescopic combination of a concave and a convex spherical mirror allows gradual adjustment of the effective focal length (≈4.2 m) and therefore of the beam size in focus at the HCF entrance. The angles of incidence on the two mirrors can also be adjusted such that the astigmatism introduced by the first mirror is compensated by that introduced by the second mirror. To ensure the system’s long-term stability, we maintain optimal coupling into the fiber with a 4D beam stabilization system (Aligna, TEM Messtechnik, Hannover, Germany), locking the near- and far-field positions by active feedback to two piezo-driven mirror mounts. After the HCF, the pulses are compressed by a combination of two fused silica wedges and a set of six pairs of highly dispersive (−40 fs^2^ each) double-angle chirped mirrors (PC70, Ultrafast Innovations).

The home-built f-to-2f spectrometer does not include a spectral broadening stage, but includes a β barium borate crystal for frequency doubling, a polarizer for projecting the second harmonic and fundamental onto the same polarization direction, and a fiber-coupled spectrometer (SP3-USB, Thorlabs, Newton, New Jersey). The analysis of the spectra and generation of the error signal is implemented in the APS800 software by Menlo Systems (Garching, Germany).

For the d-scan measurement, the full laser beam is sampled by an insertable uncoated fused silica wedge and sent into the commercial d-scan device (Sphere Ultrafast Photonics, Porto, Portugal), where it is focused into a thin β barium borate crystal for frequency doubling. The second harmonic spectrum is measured for a range of insertions of two fused silica wedges. An iterative algorithm then reconstructs the spectral amplitude and phase of the laser pulse in focus, providing a complete temporal characterization of the laser pulse at best compression.

The pulse energy is measured shot-by-shot by a pyroelectric energy meter (QE50SP-S-MT-D0, Gentec-EO, Québec, Canada).

## Data Availability

The raw data that support the findings of this article are available from the corresponding authors upon reasonable request.
